# Expression of MuRF1 or MuRF2 is essential for the induction of skeletal muscle atrophy and dysfunction in a murine pulmonary hypertension model

**DOI:** 10.1186/s13395-020-00229-2

**Published:** 2020-04-27

**Authors:** Thanh Nguyen, T. Scott Bowen, Antje Augstein, Antje Schauer, Alexander Gasch, Axel Linke, Siegfried Labeit, Volker Adams

**Affiliations:** 1 University Clinic of Cardiology, Heart Center Leipzig, Leipzig, Germany; 2 School of Biomedical Sciences, University of Leeds, Leeds, UK; 3 Laboratory of Molecular and Experimental Cardiology, TU Dresden, Heart Center Dresden, Dresden, Germany; 4 Medical Faculty Mannheim, University of Heidelberg, Heidelberg, Germany; 5 Myomedix GmbH, Neckargemünd, Germany

**Keywords:** Cardiac cachexia, Pulmonary hypertension, Muscle atrophy, Myofibrillar proteins, MuRF1 and MuRF2, Muscle energy metabolism

## Abstract

**Background:**

Pulmonary hypertension leads to right ventricular heart failure and ultimately to cardiac cachexia. Cardiac cachexia induces skeletal muscles atrophy and contractile dysfunction. MAFbx and MuRF1 are two key proteins that have been implicated in chronic muscle atrophy of several wasting states.

**Methods:**

Monocrotaline (MCT) was injected over eight weeks into mice to establish pulmonary hypertension as a murine model for cardiac cachexia. The effects on skeletal muscle atrophy, myofiber force, and selected muscle proteins were evaluated in wild-type (WT), MuRF1, and MuRF2-KO mice by determining muscle weights, in vitro muscle force and enzyme activities in soleus and tibialis anterior (TA) muscle.

**Results:**

In WT, MCT treatment induced wasting of soleus and TA mass, loss of myofiber force, and depletion of citrate synthase (CS), creatine kinase (CK), and malate dehydrogenase (MDH) (all key metabolic enzymes). This suggests that the murine MCT model is useful to mimic peripheral myopathies as found in human cardiac cachexia. In MuRF1 and MuRF2-KO mice, soleus and TA muscles were protected from atrophy, contractile dysfunction, while metabolic enzymes were not lowered in MuRF1 or MuRF2-KO mice. Furthermore, MuRF2 expression was lower in MuRF1KO mice when compared to C57BL/6 mice.

**Conclusions:**

In addition to MuRF1, inactivation of MuRF2 also provides a potent protection from peripheral myopathy in cardiac cachexia. The protection of metabolic enzymes in both MuRF1KO and MuRF2KO mice as well as the dependence of MuRF2 expression on MuRF1 suggests intimate relationships between MuRF1 and MuRF2 during muscle atrophy signaling.

## Background

Skeletal muscle mass adapts rapidly to activity by either activating hypertrophic or atrophic pathways. Muscle atrophy occurs as a result of changes in the balance between anabolic and catabolic processes and in many clinical conditions, like chronic heart failure [1–3], limb immobilization [4, 5], mechanical ventilation [6, 7], sepsis [8], diabetes [9], and advanced aging [10] skeletal muscle mass is lost, leading to muscle weakness, inactivity and increased mortality. The activation of both the autophagic/lysosomal proteolysis and the ubiquitin proteasome system (UPS) are recognized to play important roles in the protein breakdown. Especially the UPS system and relevant ubiquitin E3-ligases are discussed as potential targets to modulate skeletal muscle atrophy. Performing transcript profiling in several atrophy models identified MuRF1 and MAFbx as ubiquitin E3 ligases only expressed in heart and skeletal muscle [11]. MuRF1 belongs to a family of MuRF proteins consisting of MuRF1, MuRF2 and MuRF3 [12]. MuRF1 knockout animal’s exhibit resistance towards the development of skeletal muscle atrophy [11, 13] and when subjected to chronic pressure overload the animals developed massive cardiac hypertrophy [14]. MuRF2 seems to be involved in sarcomere formation [15] and intracellular signaling in cardiomyocytes by decreasing serum response transcription factor (SRF) during mechanical inactivity [16]. Furthermore, MuRF2 in mononuclear cells attenuates LPS-induced macrophage activation by inhibiting the generation of inflammatory cytokines [17]. MuRF3 binds to microtubules helping to develop a network resistant to depolarization [18], plays a role in myosin protein quality control [19] and protects against diabetic cardiomyopathy [20].

Studies analyzing the myocardium of knockout (KO) animals suggest that MuRF1 and MuRF2 play a redundant role in regulating developmental physiologic hypertrophy [21]. Synergistic cooperation between MuRF1 and MuRF2 is further supported by the observation that MuRF1/MuRF2 double KO animals present a fulminant cardiac phenotype (74% early postnatal lethality with acute heart failure), whereas single knockout animals are healthy with normal life span and myocardial functionality [22]. This cooperation among MuRFs and with other atrogenes like MAFbx was also documented in MuRF1 KO mice undergoing denervation [23] or aging [24], where a significant upregulation of MAFbx was observed when compared to wild-type animals. A molecular explanation for the cooperation between MuRF1 and MuRF2 may be their shared recognition of 35 or more protein targets [22]. These cooperative effects of MuRF1 and MuRF2 are mainly shown in the myocardium but less data are available for skeletal muscle remodeling in experimental heart failure. Therefore, the aim of the present study was to induce heart failure in MuRF1 and MuRF2 knockout mice and compare the development of muscle atrophy and dysfunction to wild-type littermates.

## Methods and materials

### Animals and study design

The mice used in this study are all on a clean C57/BL6 background. Details on the gene inactivation of MuRF1 and MuRF2 are described in Witt et al. [22]. To induce cardiac cachexia monocrotaline (MCT) was subcutaneously injected weekly at a concentration of 600 mg/kg into either C57/BL6 (WT, *n* = 9), MuRF-1 (*n* = 11), and MuRF-2 (*n* = 9) knockout animals for 8 weeks. Control animals of each group received the same volume of saline (C57/Bl6 *n* = 12; MuRF-1^−/−^*n* = 11; MuRF-2^−/−^*n* = 11). Body weight was recorded every week for each animal. Animals were exposed to identical conditions under a 12:12 h light/dark cycle with food and water provided ad libitum. Mice were sacrificed following deep anesthetization with i.p. administration of fentanyl (0.05 mg/kg), medetomidine (0.5 mg/kg), midazolam (5 mg/kg), and ketamine (100 mg/kg). At sacrifice, the heart and lungs were dissected, cleaned, blotted dry, and weighed, with the heart fixed in 4% PBS-buffered formalin. The left tibialis anterior (TA) and soleus (SO) muscle were dissected, weighed, and fixed in 4% PBS-buffered formalin, while the remaining muscle portions were immediately frozen in liquid N_2_ for molecular analysis. Muscle wet weights were normalized to tibia length, which allowed a fair comparison of relative changes in muscle mass between all groups due to differences in body weight.

All experiments and procedures were approved by the local Animal Research Council, University of Leipzig and the Landesbehörde Sachsen (TVV 40/16).

### Contractile function

The SO of the right leg was dissected to allow in vitro contractile function to be assessed using a length-controlled lever system (301B, Aurora Scientific Inc., Aurora, Canada), as previously described [25, 26]. Briefly, a muscle bundle was mounted vertically in a buffer-filled organ bath (~ 22 °C), set at optimal length, and after 15 min was stimulated over a force-frequency protocol between 1 and 300 Hz (600 mA; 500 ms train duration; 0.25 ms pulse width). Force (N) was normalized to muscle cross-sectional area (CSA; cm^2^) by dividing muscle mass (g) by the product of *L*_o_ (cm) and estimated muscle density (1.06), which allowed specific force in N/cm^2^ to be calculated.

### Tissue analyses

#### Histology

The heart (medial section), the SO, and TA muscle were embedded in paraffin; 3 μm sections were obtained, which where mounted on slides and stained with hematoxylin and eosin. Sections were than captured as images on a computer connected to a microscope and subsequently evaluated using Analysis software (Analysis 3.0, Olympus Soft Imaging Solutions GmbH, Münster, Germany). As recently described [25], RV wall thickness (in μm) was determined from the mean of 10 individual measurements distributed along the free ventricular wall, while mean fibre CSA (in μm^2^) of the soleus and TA was evaluated after assessment of approximately 300–500 fibers per animal.

### Western blot analysis

For western blot analyses, frozen TA was homogenized in Relax buffer (90 mmol/L HEPES, 126 mmol/L potassium chloride, 36 mmol/L sodium chloride, 1 mmol/L magnesium chloride, 50 mmol/L EGTA, 8 mmol/L ATP, 10 mmol/L creatine phosphate, pH 7.4) containing a protease inhibitor mix (Inhibitor mix M, Serva, Heidelberg, Germany), sonicated, and centrifuged at 16,000x*g* for 5 min. Protein concentration of the supernatant was determined (BCA assay, Pierce, Bonn, Germany) and aliquots (5–20 μg) were separated by SDS-polyacrylamide gel electrophoresis. Proteins were transferred to a polyvinylidene fluoride membrane (PVDF) and incubated overnight at 4 °C with the following primary antibodies: MuRF1 (1/1000, Abcam, Cambridge, UK), MuRF2 (1:1.600, Myomedix GmbH, Neckargemünd, Germany). Membranes were subsequently incubated with a horseradish peroxidase-conjugated secondary antibody and specific bands visualized by enzymatic chemiluminescence (Super Signal West Pico, Thermo Fisher Scientific Inc., Bonn, Germany) and densitometry quantified using a 1D scan software package (Scanalytics Inc., Rockville, USA). Blots were then normalized to the loading control GAPDH (1/30000; HyTest Ltd, Turku, Finland). All data are presented as fold change relative to control.

#### Enzyme activity measurements

TA was homogenized in Relax buffer and aliquots were used for enzyme activity measurements. Enzyme activities for citrate synthase (CS, EC 2.3.3.1), creatine kinase (EC 2.7.3.2), and malate dehydrogenase (EC 1.1.1.37) were measured spectrophotometrically as described in detail [27, 28]. Enzyme activity data are presented as the fold change vs. control.

#### Statistical analyses

Data are presented as mean ± SEM. Unpaired *t* test was used to compare groups, while two-way repeated measures ANOVA followed by Bonferroni post hoc test was used to assess contractile function (GraphPad Prism). Significance was accepted as *p* < 0.05.

## Results

### Comparison of cachexia response to MCT stress in WT, MuRF1, MuRF2 KO mice

**Fig. 1:**
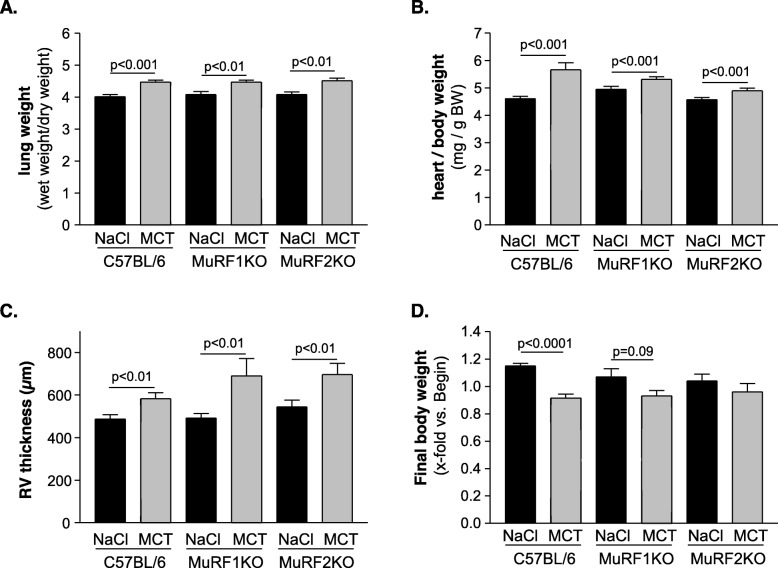
Physical characteristics of NaCl- or monocrotaline (MCT)-treated C57BL/6 wild-type animals or MuRF1 and MuRF2 knockout animals. When compared to the NaCl-treated animals, the administration of MCT to the animals had significant effects on final body weight (**a**), lung weight (**b**), heart weight (**c**), and the thickness of the right ventricle (**d**) independent of the phenotype. Data are presented as mean ± SEM

Weekly injections of MCT into WT mice are suitable to establish a chronic cardiac cachexia condition as previously described (see [25, 29]): MCT treatment of WT animals for 8 weeks resulted in increased lung weight (Fig. 1a), increased heart weight (Fig. 1b), and right ventricular hypertrophy (Fig. 1c; *p* for all groups < 0.01). Cachexia was evident after 8 weeks in WT mice with regards to their whole body weights: While control animals increased body weights by 15%, MCT mice developed a 9% reduction in body weights during the 8-week study period (Fig. 1d, *p* = 0.001). Lung and heart tissues in MuRF1 and MuRF2 KO mice responded to MCT injection similar as WT mice, and total lung and heart eights, as well as RV thickness were augmented at week 8 (Fig. 1a–c). However, the effects on body weights differed in the WT, MuRF1, and MuRF2 KO groups: MuRF1^−/−^ animals lost 7% of body weight (*p* = 0.09 MCT vs. NaCl treatment), whereas MuRF2^−/−^ lost 4% (no statistical significance when compared to NaCl-treated counterparts; Fig. 1d).

### TA and SO muscles differ in their response to MCT in WT, MuRF1 and MuRF2 KO mice

**Fig. 2:**
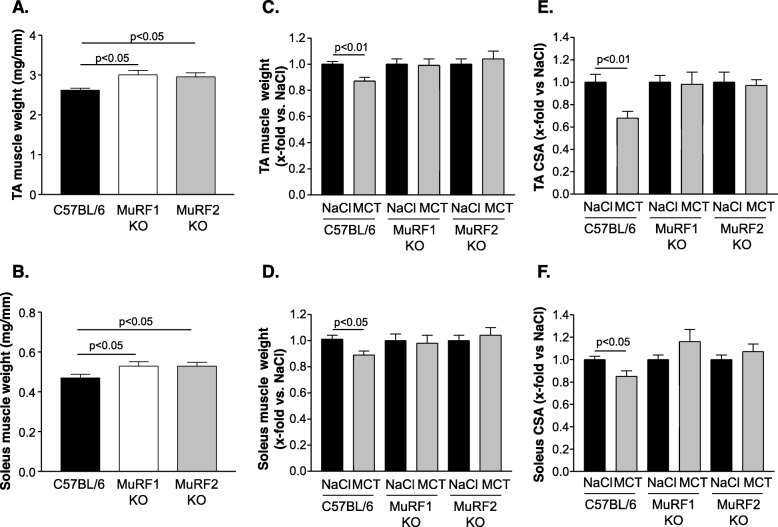
Skeletal muscle wet weight (normalized to tibia length) and cross-sectional area for soleus and tibialis anterior (TA). Muscle wet weight for the TA (**a**) and soleus (**b**) is significantly increased in both knockout animals (MuRF1KO and MuRF2KO) when compared to C57BL/6 mice. Administration of monocrotaline (MCT) to the animals resulted in a reduced muscle wet weight of the TA (**c**) and soleus muscle (**d**) in the C57BL/6 animals, whereas MCT had no effect on muscle wet weight in the MuRF1KO and MuRF2KO animals (**c**, **d**). In addition, also the cross-sectional area (CSA) of TA (**e**) and soleus (**f**) was reduced in C57BL/6 whereas this reduction was not seen in MuRF1KO and MuRF2KO mice. Data are presented as mean ± SEM

Quantifying muscle weight of the tibialis anterior (Fig. 2a) and soleus muscle (Fig. 2b) in wild-type and in MuRF1KO and MuRF2KO mice, a significant higher muscle weight was evident in MuRF1KO and MuRF2KO mice when compared to C57BL/6 animals. In accordance with our recent study [25], the whole body weight losses of WT mice underlies a progressing muscle atrophy during the eight week MCT treatment. Different degrees of muscle weight losses are present in TA and SO: while TA and SO lost about 10% total wet weights (Fig. 2c, d) (TA 10% loss, soleus 11% loss), the fiber cross-sectional area (CSA) were even markedly lower (TA 32% lowered CSA; SO 15% lowered CSA) (Fig. 2e, f). In contrast, inactivation of either MuRF1 or MuRF2 protected mice from MCT induced atrophy features both with regards to muscle wet weight (Fig. 2c, d) and fiber CSA (Fig. 2e, f). No significant differences between the NaCl and MCT groups were observed in any of the MuRF1KO and MuRF2KO SO or TA muscle measures.

### MCT-induced SO muscle force depletion is prevented by MuRF1 or MuRF2 inactivation

**Fig. 3:**
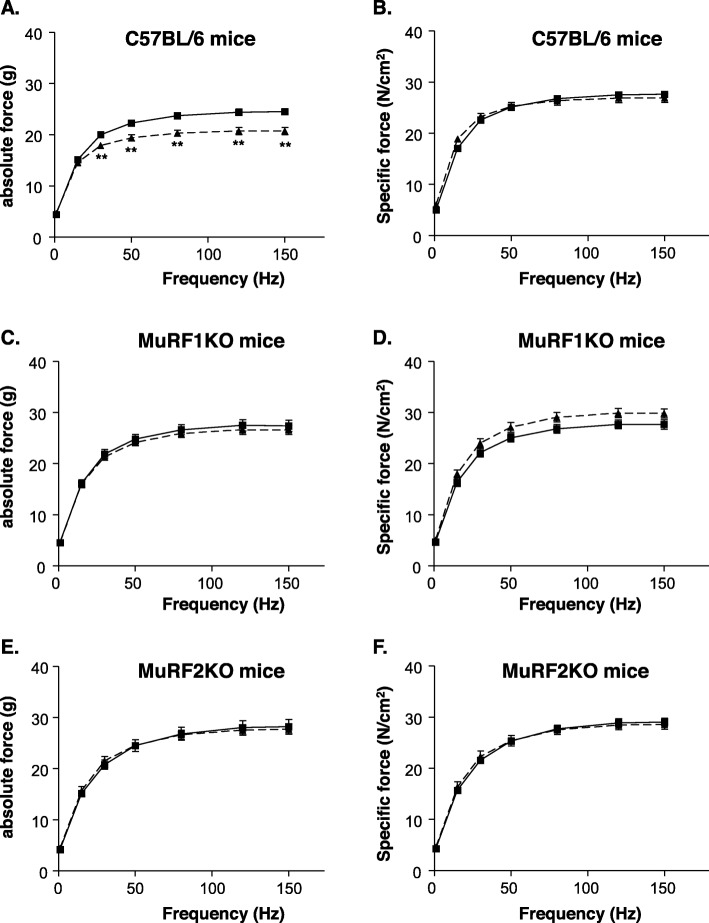
In vitro skeletal muscle function of the soleus muscle determined in C57BL/6 (**a**, **b**), MuRF1KO (**c**, **d**), and MuRF2KO (**e**, **f**) animals. Muscle force is shown as absolute force (**a**, **c**, **e**) and as specific force (**b**, **d**, **f**). Monocrotaline significantly impaired absolute muscle force only in C57BL6 animals (**a**) but not in MuRF1KO (**c**) and MuRF2KO mice (**e**). Monocrotaline had no effect on muscle specific force. Data are presented as mean ± SEM. Control animals are depicted in black squares and solid line whereas MCT-treated animals are shown in triangles and dotted lines

Next, we compared contractile properties of isolated muscle bundles from MCT-stressed and control mice by our force-frequency protocol (see “Methods” section above). Consistent with our earlier results [25], treatment of C57Bl/6 mice with MCT for 8 weeks resulted in a 16% loss of absolute SO myofiber force (Fig. 3a; *p* < 0.01). However, no change was apparent after normalization to muscle mass in terms of specific myofiber force (Fig. 3b).

In vitro comparisons of SO muscle contractility between non-MCT-treated C57BL/6, MuRF1KO and MuRF2KO mice revealed a significant higher absolute force in both knockout strains (C57BL/6 24.5 ± 0.6 g; MuRF1KO 27.6 ± 1.1 g *p* < 0.05 vs. C57BL/6; MuRF2KO 28.2 ± 1.4 g *p* < 0.05 vs. C57BL6). However, after normalization to muscle mass, specific force was not different the different strains (C57BL/6 27.7 ± 0.5 N/cm^2^; MuRF1KO 27.8 ± 0.9 N/cm^2^; MuRF2KO 28.5 ± 0.6 N/cm^2^). Intriguingly, MCT treatment for 8 weeks in did not have an impact on absolute or specific muscle forces in both MuRF1KO (Fig. 3c, d) and in MuRF2KO mice (Fig. 3e, f).

### Impact of MCT treatment on atrophy-associated protein expression

Next, during MCT stress we assessed the protein expression of MafBx and MuRF1 (two key atrogin factors). We included MuRF2 as its gene inactivation also protect from atrophy and wasting (see Figs. 1, 2, and 3).

**Fig. 4:**
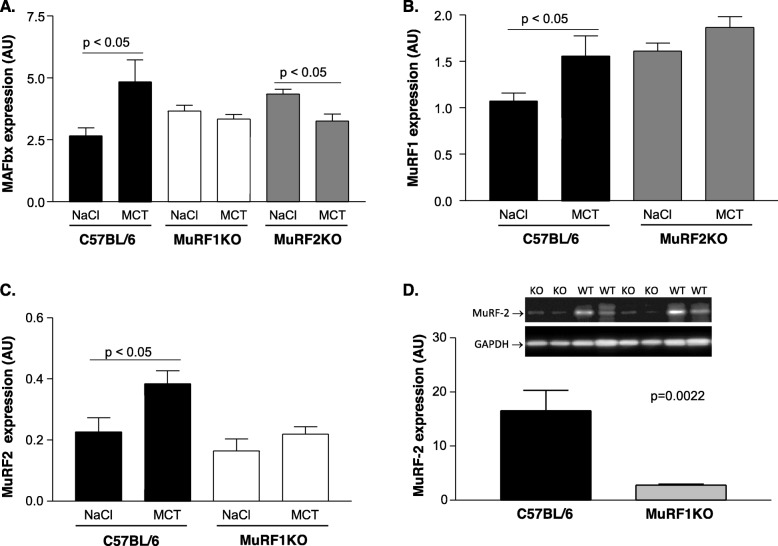
Protein expression of atrophy related proteins in the TA muscle C57BL/6, MuRF1KO, and MuRF2KO mice. MAFbx (**a**), MuRF1 (**b**), and MuRF2 (**c**) protein expression was quantified in NaCl- or monocrotaline-treated animals. To test for cooperativity between MuRF1 and MuRF2, MuRF2 expression was assessed also in C57BL6 MuRF1KO mice (**d**). Data are presented as mean ± SEM and representative blots are depicted. In the representative blot KO = MuRF-1 KO mice; WT = C57BL/6 mice

The treatment of C57BL/6 mice with MCT for a period of 8 weeks resulted in a significant increased expression of MafBx (Fig. 4a), MuRF1 (Fig. 4b), and MuRF2 (Fig. 4c). This MCT-induced upregulation of these atrogenes was not seen in MuRF1 and MuRF2KO animals. Interestingly, even a downregulation of MafBx was noted in MuRF2KO animals when treated with MCT (Fig. 4a). Finally, we tested if the expression of MuRF1 influences the expression of MuRF2. For this, we determined the expression of MuRF2 in TA muscle from MuRF1KO animals. Intriguingly, gene inactivation of MuRF1 also markedly lowered the expression of MuRF2 (see Fig. 4d for comparison of MuRF2 levels in TA of MuRF1 KO and C57BL/6 WT animals).

### Depletion of energy delivering enzymes by MCT treatment and rescue from this by MuRF1 and 2 gene inactivation

**Fig. 5:**
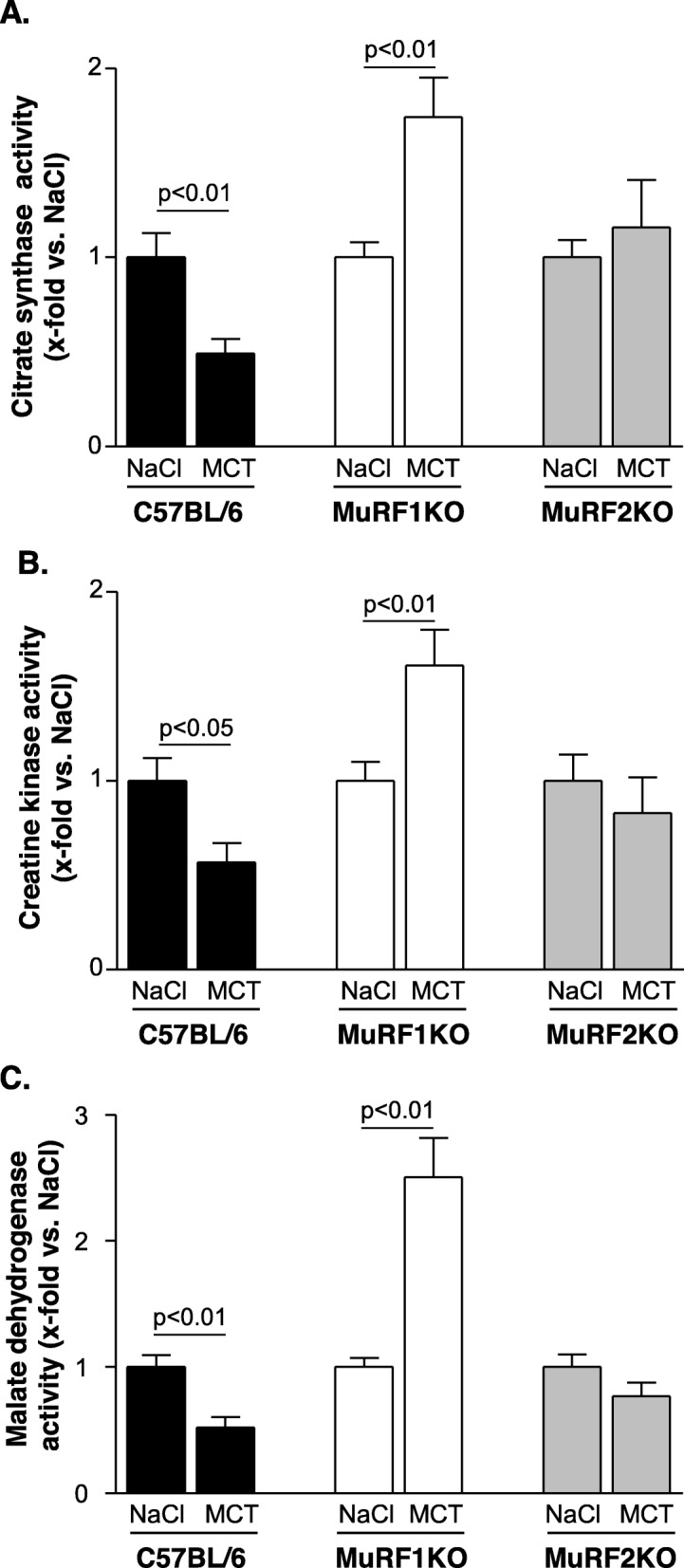
Enzymatic activity of citrate synthase (**a**), creatine kinase (**b**), and malate dehydrogenase (**c**) in the TA muscle C57BL/6, MuRF1KO, and MuRF2KO mice treated either with NaCl or monocrotaline. Data are presented as mean ± SEM

In our recent study [25] on MCT induced cardiac cachexia, we noted a depletion of enzymatic activities that generate ATP in myocytes. We therefore determined again the activity of these enzymes under MCT-stress but included also muscle extracts from MuRF1 and 2 KO mice. Consistent with our recent study [25], treatment of C57BL6 animals with MCT resulted in reduced enzyme activities for CS (Fig. 5a), creatine kinase (Fig. 5b), and MDH (Fig. 5c). In contrast, MuRF1^−/−^ animals showed an upregulation of these enzymes that are connected to mitochondrial energy metabolism in muscle. In MuRF2^−/−^ animals, MCT did not alter enzyme activities of CS, CK, and MDH in TA muscles (Fig. 5a–c).

## Discussion

Skeletal muscle atrophy occurs frequently in a variety of diseases, including tumor, chronic heart failure, diabetes, sepsis, and mechanical ventilation, contributing to a reduced muscle function and reduced quality of life. The understanding of molecular mechanisms and the relevance of specific proteins for the development of muscle dysfunction/muscle atrophy is essential for developing effective treatment strategies. In the present study we investigated in mouse models the roles of MuRF1 and MuRF2 for the development of muscle atrophy and muscle dysfunction in a right ventricular heart failure setting that mimics human heart failure during chronic pulmonary hypertension. The results of the present study can be summarized as follows: (1) the development of heart failure is associated with muscle atrophy and muscle dysfunction (loss of absolute force). This is not observed in MuRF1KO and MuRF2KO^−^ animals. (2) Muscle mass is already higher in the MuRF1KO and MuRF2KO animals when compared to WT mice independent of heart failure induction, (3) muscle atrophy induced by MCT goes along with the activation of MuRF1, MuRF2, and MAFbx, and a downregulation of enzymes involved in mitochondrial energy production and energy transfer, (4) the expression of MuRF1 influences also the expression of MuRF2.

### Importance of MuRF1 and MuRF2 for muscle atrophy in cardiac cachexia

Cardiac cachexia and the development of heart failure are often associated with skeletal muscle atrophy, being an independent prognostic marker for survival [30–33]. Therefore, understanding the molecular pathways activated and resulting in muscle atrophy are potential targets to develop specific treatment strategies to fight muscle loss and modulate morbidity and mortality. Differential transcriptional profiling has identified MuRF1 and MAFBx as markers for muscle atrophy [11, 34] and their genetic deletion resulted in muscle sparing following hind-limb unloading [35], denervation [11], or glucocorticoid treatment [36]. In the present study, we used monocrotaline to induce muscle atrophy. The application of monocrotaline to mice resulted in the development of pulmonary hypertension and right ventricular hypertrophy, evident by the increased thickness of the right ventricular wall. Inducing cardiac cachexia in C57Bl6 mice resulted in muscle atrophy as described in the current literature [25, 29, 37]. Giving monocrotaline to either MuRF1 or MuRF2 knockout animals this induction of muscle atrophy was not evident. This clearly shows for the first time that not only MuRF1 is essential for the induction of muscle atrophy, but also the expression of MuRF2 is critical for the development of muscle atrophy. Furthermore there seems to exist a cooperation or a cross-talk between MuRF1 and MuRF2 since the deletion of MuRF1 resulted, without induction of muscle atrophy, in a lower expression of MuRF2. This is in line with a recent observation by Silva et al. describing that MuRF1 directed siRNAs also knock-down expression of MuRF2 mRNA expression in cultured myotubes [38]. These findings point to an intimate connectivity between both MuRF1 and MuRF2.

The relevance of MuRF1 and MuRF2 for modulating muscle mass and muscle function is furthermore supported by our observation that the absolute muscle force is significantly higher in the MuRF1 and MuRF2 knockout animals. The molecular pathways controlled by MuRF1/2 leading to muscle atrophy therefore warrant more studies with regard to cooperativity and their signaling interrelationships: While MuRF1 ablation has been extensively studied in the context of myofibrillar protein degradation, MuRF2 was implicated in nuclear strength-regulated transcription [16]. Our data here point to joint roles in energy metabolism as another important pathway affected by both MuRF1 and MuRF2. Interestingly, the MCT-induced reduction of enzymes involved in mitochondrial energy production is different in MuRF1 and MuRF2 KO animals. This may point towards an interesting divergence in the mechanisms underlying the actions of MuRF1 and MuRF2 in this cachexia model for inducing muscle dysfunction. The different role with respect to enzymes involved in energy production is supported by the observation by Willis and colleagues [39] who observed in MuRF1 transgenic animals (specific overexpression in the heart) a significantly reduced CK activity. Applying a yeast two hybrid screen to identify specific MuRF1 and MuRF2 targets Witt and colleagues [40] reported that mainly myofibrillar proteins are targets for both MuRF1 and MuRF2 whereas the situation for enzymes involved in energy production is less clear. Nevertheless, more research is necessary to clarify the exact role of MuRF1 and MuRF2 and their interaction in inducing muscle atrophy and muscle dysfunction.

### Clinical considerations

The results discussed here were obtained using well established and previously extensively characterized KO models for MuRF1 and 2 [22, 40]. The results of the present investigation suggest that modulating MuRF1 and/or MuRF2 expression may be an attractive approach in the future to influence the development of muscle atrophy in cardiac cachexia. Unfortunately, it will be important not to completely inhibit the activity of both MuRF1 and MuRF2, because MuRF1/MuRF2 double knockout animals display a severe phenotype including severe cardiac hypertrophy massively reduced left ventricular ejection fraction and signs of heart failure [21, 22]. A recently developed and tested MuRF1/2 inhibitor from our group prevented the development of muscle atrophy and exhibited no severe side effects and was well tolerated. One possible explanation for its “side-effect”-free action is probably due to the fact that the inhibitor was screened to inhibit the interaction of MuRF1 with its target proteins but leaving its activity intact. More emphasis seems to be warranted for further drug development or chemical modulation of the described small molecule and for testing in other models of muscle wasting.

## Conclusion

In the present study, we show for the first time that in addition to MuRF1, inactivation of MuRF2 also provides a potent protection from peripheral myopathy and skeletal muscle dysfunction in cardiac cachexia. The protection of metabolic enzymes in both MuRF1KO and MuRF2KO mice as well as the dependence of MuRF2 expression on MuRF1 suggests intimate relationships between MuRF1 and MuRF2 during muscle atrophy signaling. These results also suggest that the development of a MuRF inhibitor to prevent the development of muscle atrophy should target MuRF1 as well as MuRF2.

## Data Availability

The datasets used and/or analyzed during the current study are available from the corresponding author on reasonable request.

## Abbreviations

MuRF1: Muscle ring finger protein 1
MuRF2: Muscle ring finger protein 2
MAFbx: Muscle atrophy F-box
MCT: Monocrotaline
CSA: Cross-sectional area
